# A Lever-Type Method of Strain Exposure for Disk F-Shaped Torque Sensor Design

**DOI:** 10.3390/s20020541

**Published:** 2020-01-19

**Authors:** Ran Shu, Zhigang Chu, Hongyu Shu

**Affiliations:** 1 State Key Laboratory of Mechanical Transmissions, Chongqing University, Chongqing 400044, China; ranshu@cqu.edu.cn (R.S.); zgchu@cqu.edu.cn (Z.C.); 2 College of Automotive Engineering, Chongqing University, Chongqing 400044, China

**Keywords:** torque sensor, disk F-shaped, strain exposure, lever-type

## Abstract

Disk-shaped torque sensors are widely used in robotic joints and wheel driving. However, in terms of conventional spoke-type geometries, there is always a trade-off between sensitivity and stiffness, because their strain exposure depends upon a bending deformation mode which causes strain nonuniformity. This paper presents a lever-type method of strain exposure that performs a uniaxial tension and compression deformation mode to optimize the strain uniformity and improve the trade-off. Moreover, on the basis of this approach, the proposed disk F-shaped torque sensor enjoys has axial thinness, easy installation of strain gauges and flexible customization. The simulation and experimental results have validated the basic design idea.

## 1. Introduction

Strain gauges invariably sense the normal-strain exposed on the surface of an elastic body and are commonly used for mechanical measurement. Particularly in the case of geometric design of strain-gauge-based torque transducers, it is desirable to transform a measured moment into a uniform normal-strain exposure. Some commercial shaft torque sensors, which measure strain exposed at 45° and 135° to the cylinder neutral-axis with a torsional deformation mode, have been specially used in laboratory testing for a long time [1,2,3,4,5]. Many disk torque sensors developed into spoke-type structures, instead, expose strain on the transverse or lateral spoke-surface with a bending deformation mode [6,7,8,9,10,11,12,13,14,15,16,17,18,19,20,21,22,23,24]. Recently, owing to axial thinness and low sensitivity to non-torsional components, they have been extensively integrated in robotic joints, wheel driving and intelligent products.

However, there is a trade-off between stiffness and sensitivity when these sensors are put into practical applications. A six-axis force/torque sensor reported by Chao et al. [8] increases the sensitivity with an excellent decoupled calibration matrix. An appealing square-cut torque sensor presented by Khan et al. [14], has enhanced the sensitivity without reducing its linearity and symmetry. Further, this scalable sensor can be implemented in a robotic joint. The ultra-low-cost custom torque sensor proposed by Ubeda et al. [18] achieves a good performance in terms of sensitivity and stiffness, meanwhile it is inexpensive and can be easily machined. The trade-off still exists, because there are two conflicting requirements.

1. High stiffness is important to ensure system dynamic performance, and position control accuracy, as sensors are integrated in products.
2. Large strain is needed to increase the sensitivity and signal-to-noise (S/N) ratio of the sensor [25,26].

However, sensitivity introduces a torsion compliance, which can cause trouble when used in ultra-high precision and high bandwidth torque measurement of micro robotics [27].

Some researchers proposed advanced spoke-structures to address this challenge. An interesting hollow hexaform structure was presented by Aghili et al. [25] to expose strain on the lateral site of wings, minimizing the interaction between sensitivity and stiffness and decreasing the sensitivity for the other five force/torque components. A 4-bar linkage geometry was reported by Zhang et al. [28], which effectively amplifies the strain by a mechanical method with little impact on stiffness because it separates the sensing point and support portion.

These advanced spoke-like geometries have mitigated the trade-off to some extent, but they are still limited by a bending deformation mode. Besides, to the best of our knowledge, developing a method of strain exposure based on the deformation mode is not usually considered for trade-off optimization.

In this paper, a lever-type method of strain exposure (hereafter abbreviated as LTMSE) is presented. Unlike the above spoke-type transducers, the disk F-shaped torque sensor (hereafter abbreviated as DFTS) based on LTMSE, performs a uniaxial tension and compression deformation mode to expose uniform normal-strain. The trade-off can be further improved with a lower effect on overall stiffness by reducing the sensing area. Also, DFTS offers a compact axial-space to ease the difficulty of gluing and wiring of strain gauges, conveniently employing a full-bridge circuit to reduce temperature drift and decouple non-torsional components. Besides, it can also be easily customized and integrated.

## 2. Design and Method

### 2.1. Strain Exposure by Bending Deformation Mode

**Figure 1:**
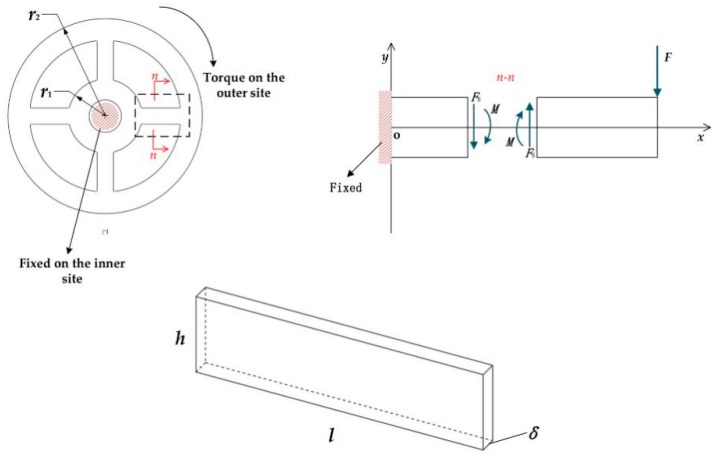
Schematic view of the spoke-like torque sensor: (**top left**) the loading and constraints on torque sensor; (**top right**) close-up view for the spoke part; (**bottom**) the parameters of the spoke.

To better illustrate the bending deformation mode, here we give an example of strain measurement in a typical spoke structure. The schematic view is shown in Figure 1.

A torsion *T* acts on the spoke-like geometry, equivalent to a loading force *F*. To calculate the sensitivity, in this part, we assume that $F\;=\;\frac{T}{{4(r}_{1}+l)\;}$, which creates the bending moment *M* and the shear force *F*_s_ on the cross-section *n*-*n*. For nullifying the *M*, based on Mohr’s theorem, the method of obtaining the strain created by only sheer force *F*_s_ (see Equation (1)) is to attach four strain gauges at l/2 of the spoke on the transverse site (two strain gauges glued in the front at ±45° to the spokes’ neutral-axis, others are correspondingly in the back) [29].

(1) $$F_{s}\;=\;\frac{T}{{4(r}_{1}+l/2)}\;$$

where $r_{1}$ is the radius of the inner ring and l is the length of spokes. Then the shear stress τ on the cross-section *n*-*n* is

(2) $$\tau \;=\;\frac{{6F}_{S}}{{h\delta }^{3}}(\frac{\delta^{2}}{4}{-y}^{2})$$

where *δ* is the thickness and h is the height of spokes. Two strain gauges (on the front) are mounted at ±45° to the spokes’ neutral-axis, i.e., *y* = 0. From Equation (2), we can further obtain

(3) $$\tau_{max}\;=\;\frac{{3F}_{s}}{2h\delta }$$

Subsequently, on the neutral surface, tensile/compressive normal stresses can be expressed as

(4) $$\sigma_{1}{\;=\;\tau }_{max}\;=\;\frac{{3F}_{s}}{2h\delta }$$

(5) $$\sigma_{2}\;=\;-\;\tau_{max\;}\;=\;-\;\frac{{3F}_{s}}{2h\delta }$$

According to the general Hooke’s law, the normal strain is given by

(6) $$\varepsilon_{1}\;=\;-\;\varepsilon_{2}\;=\;\frac{{3F}_{s}\left(1+\mu \right)}{2Eh\delta }$$

where *μ* is Poisson’s ratio and *E* is Young’s modulus. Hence, the total strain $\;\varepsilon \;=\;2\;\left(\varepsilon_{1}-\varepsilon_{2}\right)$, from four strain gauges that are electronically connected, can be defined as

(7) $$\varepsilon \;=\;\frac{3T(1+\mu )}{{2Eh\delta (r}_{1}+l/2)}$$

*F*_s_ is substituted by Equation (1). And the sensitivity is predicted by

(8) $$S\;=\;\frac{\varepsilon }{T}\;=\;\frac{3(1+\mu )}{{2Eh\delta (r}_{1}+l/2)}$$

To calculate the stiffness, we simplify the spoke geometry into the beam. To simplify the equations, in this part, we assume that the equivalent *F* = $T/{4r}_{2}$, where *r*_2_ denotes the radius of the outer ring. Then the deflection θ [30] is described as

(9) $$\theta \;=\;\frac{{Tl}^{3}}{{E\delta r}_{2}{}^{2}h^{3}}$$

It yields the stiffness

(10) $$K\;=\;\frac{T}{\theta }\;=\;\frac{{E\delta r}_{2}{}^{2}h^{3}}{l^{3}}$$

Last, we obtain the comprehensive index, representing the product of sensitivity and stiffness, expressed as

(11) $$\eta \;=\;\frac{\varepsilon }{\theta }\;=\;\frac{{3(1+\mu )h}^{2}r_{2}{}^{2}}{{2(r}_{1}{+l/2)l}^{3}}\;$$

**Figure 2:**
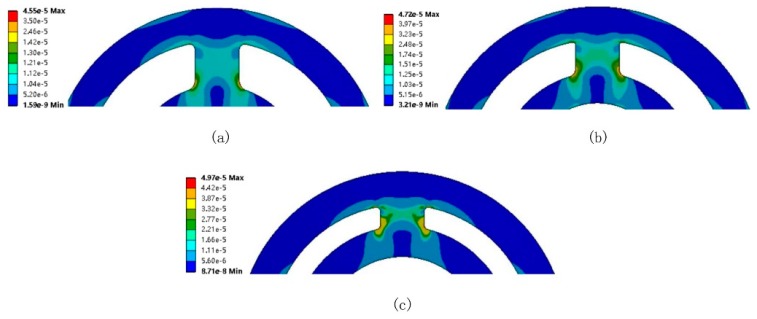
Effect of h/l on the strain distribution (equivalent strain result): (**a**) h/l = 1; (**b**) h/l = 1.5; (**c**) h/l = 2.

On account of the spoke component, the direct improvement of the trade-off can be achieved by moderately increasing *h* and decreasing *l*; i.e., by increasing h/l (see Equation (11)). But such an increment will undermine strain uniformity, as shown in Figure 2, which is tightly related to measuring error [31,32].

The increment of h/l can increase the stiffness (see Equation (10)) but it induces poor strain uniformity on the transverse/lateral site, as well as the stress concentration and boundary effect. However, decreasing  h/l to improve the strain uniformity may sacrifice stiffness. This means that the adjustment of h/l is not a good choice for the improvement of the trade-off. With respect to spoke-like geometries, if they employ a bending deformation mode to expose strain, this generally introduces poor strain uniformity. Strain nonuniformity occurs probably because the force *F* (see Figure 1) does not act through the centroid of the spoke’s cross-section, which brings about the reduction of sensitivity and resolution [30]. Moreover, such a disadvantage results from the elastomer itself and cannot be overcome by enhancing the gauge factor and amplifying the magnitude of the electric circuit. This is the reason that the extent to the improvement of the trade-off is limited. Therefore, to reconcile the conflict between sensitivity and stiffness, a new approach based on the deformation mode is needed to expose large strain on the sensing site, and retain good uniformity within small geometry size. Meanwhile, the part of elastomer not used for the strain measurement should remain robust for high stiffness.

### 2.2. LTMSE with Uniaxial Tensile/Compressive Deformation Mode

**Figure 3:**
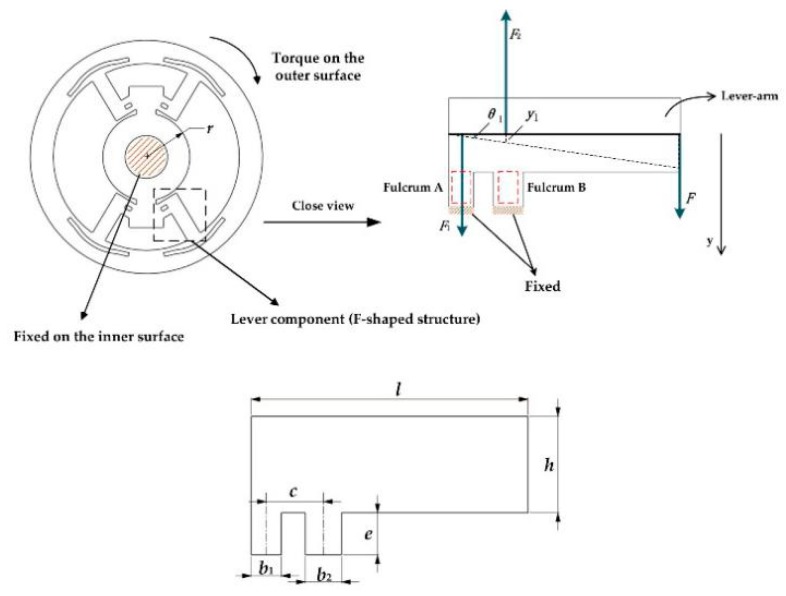
Schematic view of the disk F-shaped torque sensor (DFTS): (**top left**) the loading and constraints on DFTS; (**top right**) force analysis of the lever-arm; (**bottom**) the parameters of lever.

For mitigating the limitation of bending deformation mode, the LTMSE is proposed. The schematic view of DFTS is shown in Figure 3. Without loss of generality, it is assumed that the inner ring is fixed and the torque is loaded on the outer side with one free translational degree of freedom in the motion direction.

To calculate the sensitivity, when a torque *T* is given, the equivalent downward force *F* applies on the lever (F-shaped structure) expressed as

(12) $$F\;=\;\frac{T}{4(r+l)}\;$$

where *r* is the radius of the inner ring and *l* is the length of levers. The force *F* produces $F_{1}\;=\;F(l-c)/c$ and $F_{2}\;=\;-\mathrm{Fl}/c$; thus, the normal stress *σ*_1_ and *σ*_2_, associated with $F_{1}$ and $F_{2}$, on the fulcrum A and B are created.

(13) $$\sigma_{1}\;=\;\frac{T(l-c)}{{4b}_{1}c\delta (r+l)}\;$$

(14) $$\sigma_{2}\;=\;-\frac{Tl}{{4b}_{2}c\delta (r+l)}\;$$

where δ is the thickness, $b_{1}$ and $b_{2}$ are the width of fulcrum A and B respectively, and *c* is the distance between them. As can be seen in Figure 3, Equations (13) and (14), the fulcrum A, squared with a red dotted line, is in tension, whereas the fulcrum B is in compression. Accordingly, the normal strain on the fulcrums can be depicted by

(15) $$\varepsilon_{1\;}=\;\frac{T(l-c)}{{4Eb}_{1}c\delta (r+l)}$$

(16) $$\varepsilon_{2\;}=\;-\frac{Tl}{{4Eb}_{2}c\delta (r+l)}$$

respectively, where *E* is Young’s modulus.

Four strain gauges are fixed on four fulcrums separately (the DFTS is composed of four F-components, one F-part with two fulcrums); hence, the total strain $\;\varepsilon \;=\;2\;\left(\varepsilon_{1}-\varepsilon_{2}\right)$, from a full bridge circuit is defined as

(17) $$\varepsilon \;=\;\frac{T}{2c\delta E(r+l)}(\frac{l-c}{b_{1}}+\frac{l}{b_{2}})$$

And the sensitivity *S* is predicted by

(18) $$S\;=\;\frac{\varepsilon }{T}\;=\;\frac{1}{2c\delta E(r+l)}(\frac{l-c}{b_{1}}+\frac{l}{b_{2}})$$

To calculate the stiffness, the displacement $y_{1}\;$ of two fulcrums (see Figure 3 top right) is given by

(19) $$y_{1}\;=\left(\varepsilon_{1}-\varepsilon_{2})e\;=\;\frac{Te}{4c\delta E(r+l)}(\frac{l-c}{b_{1}}+\frac{l}{b_{2}}\right)$$

Next, we obtain the angle deflection

(20) $$\theta_{1}\;≅\;\tan\theta_{1}\;≅\;\;y_{1}/c\;=\;\frac{Te}{4c^{2}\delta E(r+l)}(\frac{l-c}{b_{1}}+\frac{l}{b_{2}})$$

The total deflection θ is mainly related to eight fulcrums (the DFTS consists of eight fulcrums), when lever-arms, inner and outer rings are treated as rigid bodies, described as

(21) $${\theta \;=\;4\theta }_{1}\;=\;\frac{Te}{c^{2}\delta E(r+l)}(\frac{l-c}{b_{1}}+\frac{l}{b_{2}})$$

where *e* is the height of fulcrums, which yields the stiffness

(22) $$K\;=\;\frac{T}{\theta }\;=\;\frac{c^{2}\delta E(r+l)}{e(\frac{l-c}{b_{1}}+\frac{l}{b_{2}})}$$

Hence, we can further obtain the comprehensive index as follows

(23) $$\eta \;=\;\frac{\varepsilon }{\theta }\;=\;\frac{c}{2e}$$

From Equation (23), the trade-off can be improved by only increasing c/e appropriately. In contrast with the spoke-shape, this simple formula is more direct and effective for the improvement of the trade-off.

Furthermore, on the disk F-geometry, the reacting forces associated with *F*_1_ and *F*_2_ on two fulcrums act through the centroid of cross-sectional area, making the normal strain very pure and irrelevant to other deformations. Note that the direction of tension/compression on fulcrums is only along that of the height *e*; in other words, two fulcrums exhibit a uniaxial tensile and compressive deformation mode. LTMSE utilizes such a deformation mode for keeping good strain uniformity and sensitivity, accordingly improving the trade-off, as further discussed in the simulation and analysis sections. In addition, fulcrums can be flexibly customized to glue strain gauges, offering compact space for wiring and electronically connected to form a full-bridge circuit which compensates the temperature drift and achieves the natural decoupling.

### 2.3. The Improvement of the DFTS

**Figure 4:**
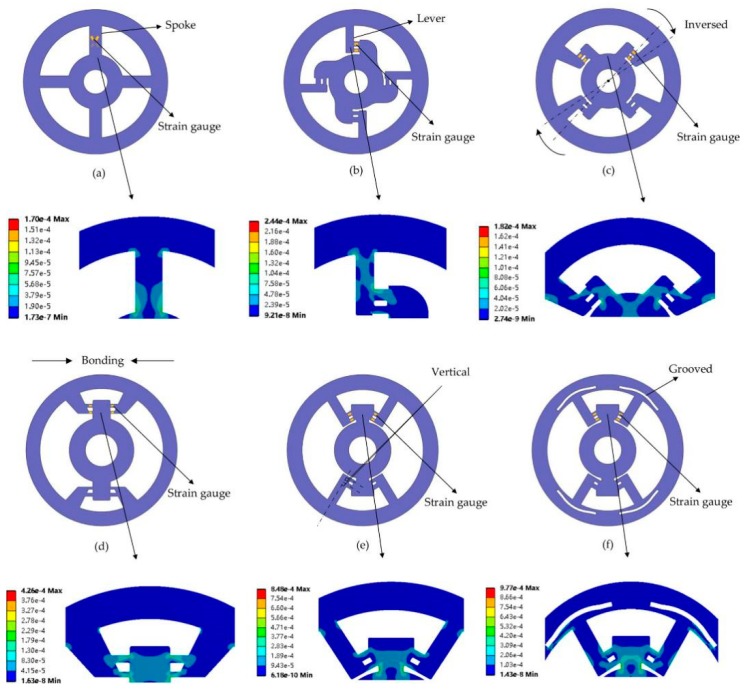
Optimization of the DFTS, shown in sequence: (**a**) hub-sprocket four spoke shape; (**b**) preliminary lever-type shape; (**c**) inversed lever-type shape; (**d**) bonding lever-type shape; (**e**) vertical F-disk shape; (**f**) prototype of DFTS.

The prototype of the DFTS has undergone several modifications for a better performance (see Figure 4). Simulation results (equivalent strain) for each structure are shown below. The location of strain gauges is denoted as a yellow solid square. Fulcrums can be specially customized for the strain gauge installation. As the method of strain exposure is highly related to structure design, the following four goals should be considered:

1. Easy installation of strain gauges;
2. Exposure of strain on primarily on the transverse surface;
3. Good strain uniformity and symmetry;
4. Serving a double purpose for sensitivity and stiffness.

Based on the four goals, the evolution is explained as follows.

The geometry modification began with a typical spoke-like shape as shown in Figure 4a. Two strain gauges were glued in the front, and the other two are in the back. With some defects mentioned in Section 2.1, this geometry was abandoned. Motivated by LTMSE, the preliminary lever-type geometry (b) is presented to expose normal strain on the transverse surface where strain gauges can be easily glued. Similarly, two strain gauges were glued in the front, and the other two in the back. The trade-off is improved because the lever-arm can be enlarged to increase the stiffness and the fulcrum can be minimized to increase the sensitivity.

To keep the same strain value on two fulcrums, a simple conjecture is that the centerline of the lever-arm should pass through the centroid, making the equivalent force *F* (see Figure 3) perpendicular to the lever-arm’s centerline. In addition, the strain gauges should be glued onto the same surface for easily connecting a full-bridge circuit. This structure (see Figure 4c) was presented to reverse two levers being axially symmetric when subjected to a clockwise or counterclockwise torsion. Four strain gauges are axially symmetrically positioned on the transverse site of the levers. Due to compact arrangement on the outer surface, this reduces the difficulty of installing strain gauges and decreases the temperature drift.

In view of the long distance between two reversed levers, which increases the difficulty of wiring strain gauges, the next structure (Figure 4d) was designed to bond pairs of axial symmetric levers to effectively utilize the space.

Nevertheless, as can be seen in Figure 4d, the direction of strain distribution is not along the height *e* of fulcrums even though strain values on two fulcrums are almost identical. In response, the elastomer (Figure 4e) was proposed to make the lever-arm perpendicular to the fulcrums, i.e., the fulcrums’ deformation should be parallel to the force *F*. This adjustment was to let the torque sensor perform a uniaxial tensile/compressive behavior.

However, the simulation result from Figure 4e indicates that the direction of strain distribution was modified yet strain values on two fulcrums become different. Our explanatory hypothesis is that the outer ring was too rigid for levers, leading to the radial displacement of the lever-arm which makes the strain on fulcrums non-uniform. Therefore, for keeping better strain uniformity, the slit of the outer ring was designed in the structure (f) to weaken the radial stiffness. Although the groove addition brings about the tangential deformation (decreases the torsion stiffness), compared with the fulcrum’s deformation, tangential deformation occurs at the outer end of the lever with less weakening effect on the overall torsion stiffness; i.e., this compromise is acceptable.

## 3. Simulation and Analysis

First, the F-shaped geometry (lever geometry), as an independent component extracted from the DFTS, was tested for strain uniformity. Second, the simulation of DFTS was carried out to evaluate whether it is symmetric and performs a tensile/compressive deformation mode for good sensitivity. Note that the DFTS, as a one-axis torque sensor, requires only pure torque measurement under any circumstance, so in the simulation section, the DFTS is only loaded by an exclusive torque M_z_. Finally, in the analysis section, parameters’ effect on the sensitivity, stiffness and comprehensive index are discussed. Within the DFTS structure, a large deformation occurs on fulcrums, which means that the stiffness on this part is weak. But a large deformation is needed to retain high sensitivity; this is the trade-off. Thus, the focus point in the analysis section is on fulcrums. There are five parameters that should be considered: b_1_, b_2_, c, e, δ; constant parameters: elastic modulus E = 200 Gpa, Poisson’s ratio μ = 0.25, *r* (the radius of the inner ring) = *l* (the length of levers) = 0.02 m.

### 3.1. Simulation

#### 3.1.1. F-shaped Component

**Figure 5:**
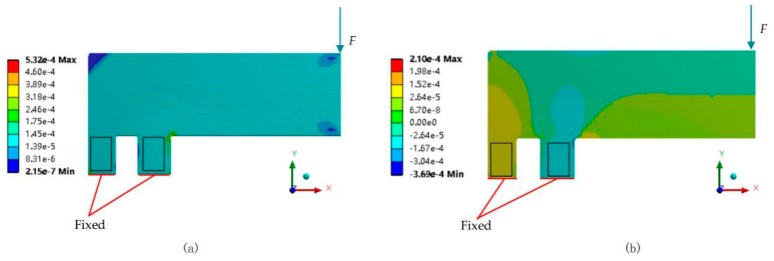
Simulation results of lever geometry: (**a**) the equivalent elastic strain result; (**b**) the normal elastic strain (y-axis) result.

To estimate the strain uniformity on the lever part and ensure the effectiveness of strain gauges’ work, ANSYS^TM^ is used to evaluate the strain direction and value according to the load. The right end of the lever-arm is loaded by a downward force (40 N) and the bottoms of the two fulcrums are fixed. Moreover, some of the right-angle area is modified to become a fillet to avoid the stress concentration. The simulation result is shown in Figure 5. The equivalent strain values of two fulcrums are almost the same as Figure 5a. It should be noted that the parameter *b*_2_ is designed to be larger than *b*_1_ ($b_{2}=\;\frac{b_{1}l}{l-c}$) for maintaining the same strain value on two fulcrums. In Figure 5b, the yellow color represents the positive strain along Y-axis and the blue color represents the negative strain, i.e., the area in the black square displays the tension or compression in one direction (y-axis), indicating that the F-shaped component exhibits a uniaxial tensile/compressive deformation mode and maintains good strain uniformity on the sensing site. Thus, strain gauges can be easily installed at this position to effectively measure the strain.

#### 3.1.2. DFTS Model

**Figure 6:**
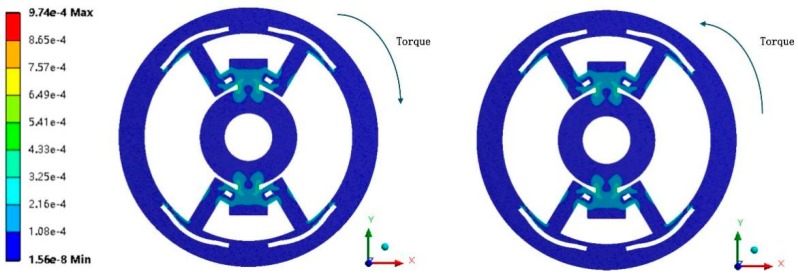
The equivalent strain result of the DFTS model: (**left**) the clockwise rotation is loaded; (**right**) the counterclockwise rotation is loaded.

To envision the behavior of DFTS, the inner ring is fixed and the outer one is loaded by a given torque *M*_z_ (10 Nm). First, the outer ring is loaded by a clockwise torsion and its strain distribution is shown in Figure 6 (left). Then the outer ring is loaded by a counterclockwise torsion (see in Figure 6 (right)). Simulation results illustrate that the strain values on fulcrums, between the left and right, remains the same; i.e., the DFTS enjoys symmetric behavior.

**Figure 7:**
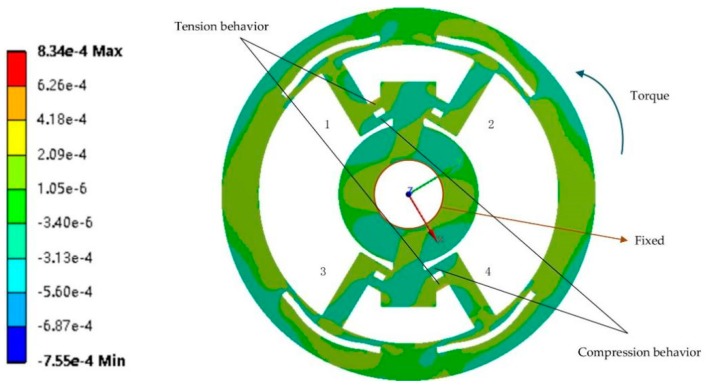
The normal strain (y-axis) result (yellow for positive strain and blue for negative strain).

Second, the deformation mode of fulcrums was tested for a pure tensile/compressive performance. Because of its symmetry, here we only chose a counterclockwise torque (10 Nm) acting on the outer ring. In Figure 7, a coordinate system is constructed to demonstrate the behavior of fulcrums. The orientation of the y-axis is parallel to that of the height *e* on fulcrums. In the simulation, F-components 1 and 4 perform a compression–tension behavior. Components 2 and 3 correspondingly have a tension–compression behavior (not shown in figure) owing to the symmetry; i.e., four F-components exhibit a uniaxial tensile/compressive deformation mode.

### 3.2. Analysis

**Figure 8:**
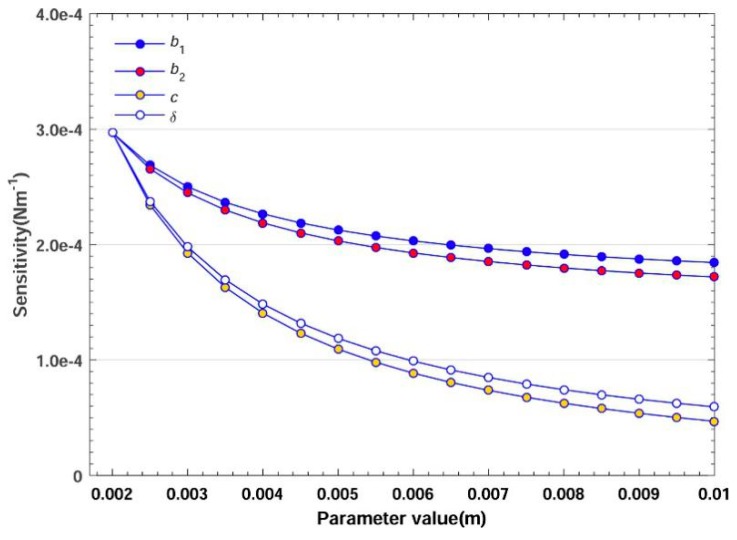
Effect of parameters on sensitivity.

**Figure 9:**
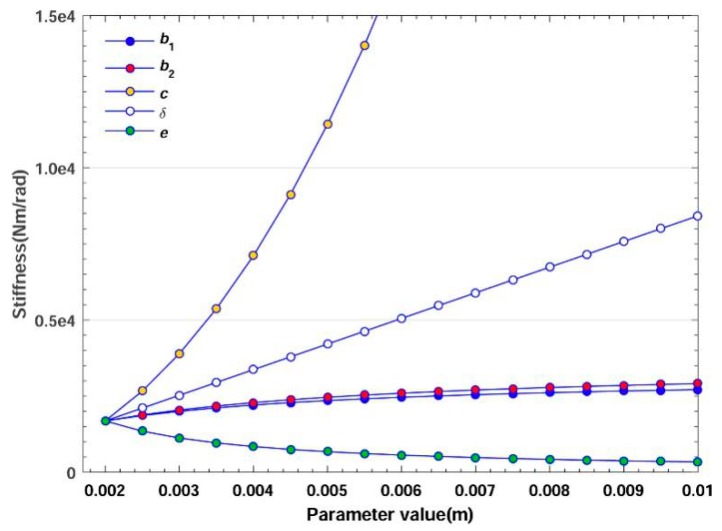
Effect of parameters on stiffness.

**Figure 10:**
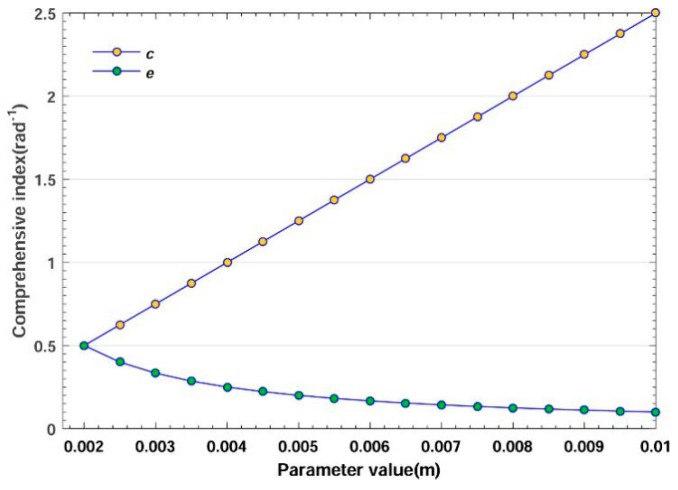
Effect of parameters on the comprehensive index.

The variation of different parameters will affect the sensitivity, stiffness and comprehensive index, as shown in Figure 8, Figure 9 and Figure 10 respectively. The parameter values are as follows: *b*_1_ = 0.002 to 0.01 m, $b_{2}$ = 0.002 to 0.01 m, *c* = 0.002 to 0.01 m, *δ* = 0.002 to 0.01 m, *e* = 0.002 to 0.01 m. When one parameter varies, the others are set as 0.002 m.

#### 3.2.1. Effect on Sensitivity

In Figure 8, the increment of $b_{1}$, $b_{2\;}$, *c* and *δ* has a negative impact on the sensitivity, yet *e* is irrelevant to the sensitivity (see Equation (18)). Within the same increment from 0.002 to 0.01 m, *c* causes the sensitivity to decrease about 6 times, whereas $b_{1}$ only causes a decrease of 1.7 times, suggesting that *c* has a greater effect. During the strain measurement, the strain gauges’ size is related to $b_{1}$, $b_{2\;}$ and *e*. So, moderately decreasing these factors will save the space and enhance the sensitivity.

#### 3.2.2. Effect on Stiffness

Figure 9 illustrates the effect on stiffness among these factors. For displaying the impact of five parameters in detail, the range of y-axis is set from 0 to 1.5 × 10^4^. The increment of $b_{1}$, $b_{2}$, *c* and *δ* has a positive impact, whereas that of *e* has a negative impact. Within the same increment from 0.002 to 0.005 m, *c* led the stiffness to rise nearly 8 times, whereas $b_{1}$ led to an increment of only 2 times, suggesting that *c* also has greater impact. As mentioned in Section 3.2.1, $b_{1}$ and $b_{2\;}$ should be decreased to maintain good sensitivity. Though this reduction has a negative impact on stiffness (see Figure 9), the effect is small compared with other factors. Furthermore, this sacrifice can be compromised by increasing the lever-arm’s height *h* and thickness. Considering that the trade-off is mainly related to fulcrums, the effect of *h* related to the lever-arm is not discussed.

#### 3.2.3. Effect on Comprehensive Index

Decreasing the interaction between sensitivity and stiffness is the goal of trade-off optimization. Comprehensive index η—the product of sensitivity and stiffness—represents the improvement of the trade-off, which is expected to reach a high value. In Figure 10, within the same increment from 0.002 to 0.01 m, *c* and *e* bring about 5 times the increment and reduction of η respectively. The parameters $b_{1}$, $b_{2\;}$ and *δ* are irrelevant to the comprehensive index (see Equation (23)). That is, in different situations, when other parameters have been determined, appropriately increasing *c*/*e* will effectively optimize the trade-off.

## 4. Experiment

**Figure 11:**
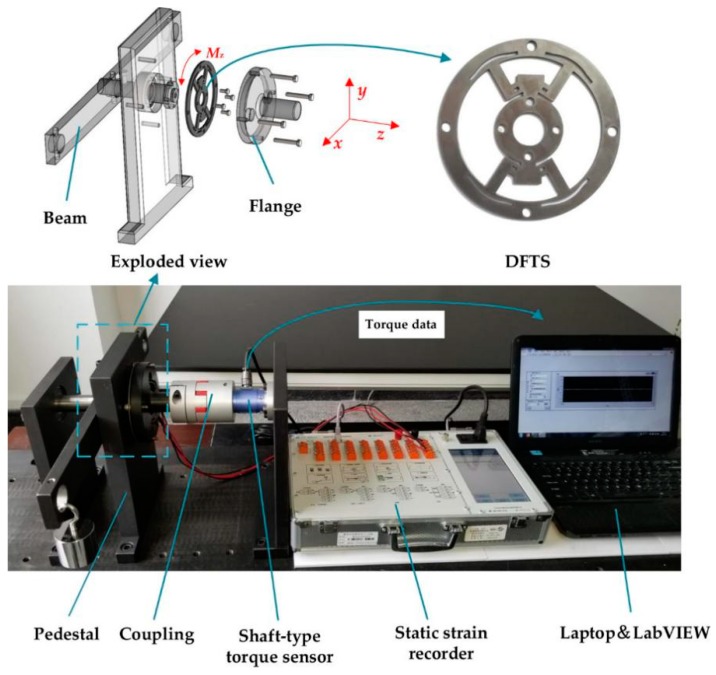
Experimental setup of strain measurement.

To test the performance of the DFTS, experiments have been carried out for obtaining the relationship between the strain and torque and the relationship between the deflection and torque. The DFTS is made of 45 stainless steel. The values of five parameters are set as follows: *b*_1_ = 0.0025 m, *b*_2_ = 0.003 m, *c* = 0.0048 m, δ = 0.003 m, *e* = 0.0035 m. DFTS requires only a pure torque measurement, so the experimental setup is under the circumstance of loading an exclusive torque *M*_z_. The experimental setup of strain measurement is shown in Figure 11.

The surface of fulcrums was initially cleaned to install strain gauges. The parameters of strain gauges are shown in Table 1. Four strain gauges were electronically connected to form a full bridge circuit. And a static strain recorder is employed to obtain the total strain from four strain gauges.

Then, the inner ring, allowed to have one free translational degree of freedom in the motion direction, was linked to the beam loaded by a mass through a bearing. Thus, a pure clockwise or counterclockwise torque was produced by placing a mass on either end of the beam. The measuring torque range of DFTS is from −10 to 10 Nm, computed by T = mgd, where *m* is the mass, *g* is the gravitational acceleration and *d* is the distance away from the center. The outer ring is connected to the shaft-type torque sensor through a flange and a coupling. The right end of this sensor is fixed through four bolts on a pedestal. This shaft-type torque sensor was used to obtain the loading torque value displayed by LabVIEW^TM^. The relationship between the strain and torque is shown in Figure 13a.

**Figure 12:**
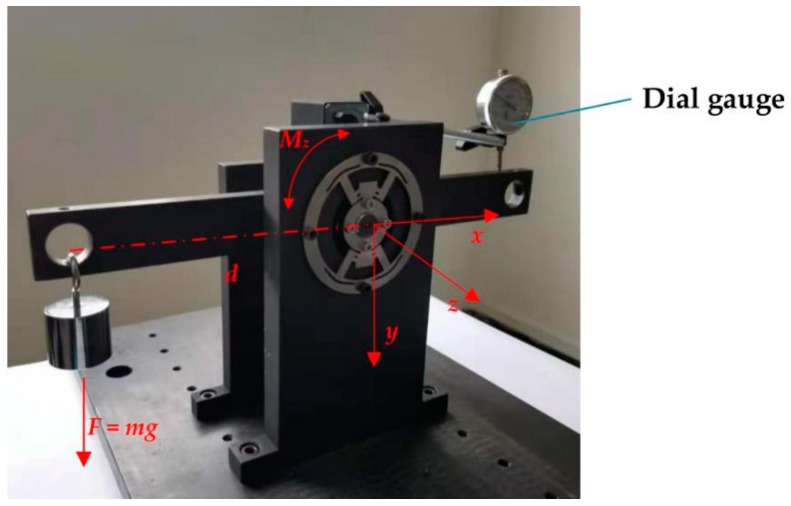
Experimental setup of deflection measurement.

Next, the setup for measuring the deflection is shown in Figure 12. The outer ring is through four bolts directly fixed on a pedestal and the inner ring is linked to the beam loaded by a mass. The dial gauge is used to record the x-axis displacement of the inner ring affected by mass variation; the distance *d* is constant. So, the angle deflection of DFTS can be expressed as θ ≅ tanθ= Δy/d.

**Figure 13:**
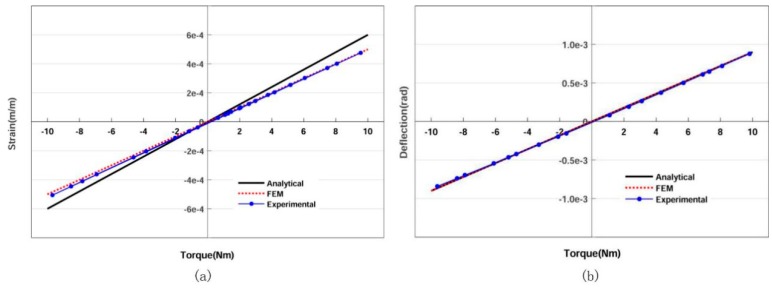
Experimental results: (**a**) relationship between torque and strain (blue line: *y* = 5.1 × 10^−5^*x* − 8.6 × 10^−6^, *R*^2^ = 0.99); (**b**) relationship between torque and deflection (blue line: *y* = 8.86 × 10^−5^*x* − 3.85 × 10^−6^, *R*^2^ = 0.99).

The relationship between the torque and deflection is shown in Figure 13b. The torque range is from −10 Nm to 10 Nm.

**Figure 14:**
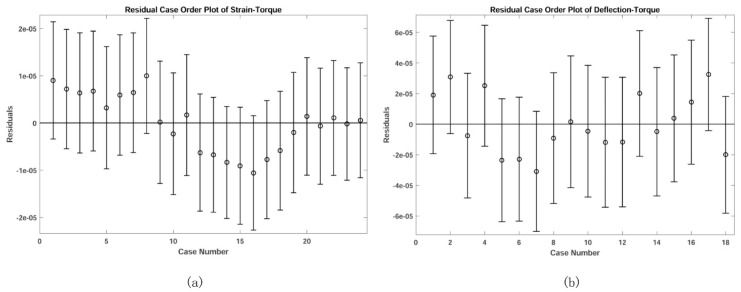
Residual analysis results: (**a**) residual case order plot for strain-torque data; (**b**) residual case order plot for deflection-torque data.

The blue dots are the experimental data and the blue line was obtained via regression. The blue line illustrates that DFTS enjoys a good linearity (linear regression coefficient $R^{2}\;$ is near to 1) and symmetry according to the payload. The consistency of comparison among experimental data, FEM result and analytical estimation suggests the feasibility of LTMSE. Then the sensitivity and stiffness of DFTS were calculated as follows: *S* = 5.1 × 10^−5^ Nm^−1^, *K* = 1.1 × 10^4^ Nm/rad. The red dotted line was computed by ANSYS^TM^ and the black line is calculated by equations. The difference between experimental and FEM results is due to the nonlinearity of strain gauges and friction among devices. Figure 14 shows the residual analysis for experimental data (no abnormal data); and the small residual suggests that the blue line could correctly fit the experimental data. 

To further test the performance of DFTS, several key parameters are shown in Table 2.

The hysteresis is 0.9% FS, mainly due to the friction among experimental devices. As can be seen in Table 2, the DFTS enjoys a good linearity and resolution. Given the DFTS is a prototype model, these factors can be further optimized in target applications which will be shown in future reports. 

## 5. Conclusions

A prototype of a DFTS based on LTMSE is proposed in this paper. Firstly, the uniform normal strain on the transverse surface is exposed with LTMSE by a uniaxial tension and compression deformation mode. Compared with the traditional measurement in spoke-like torque sensors, this deformation mode can enhance the strain uniformity due to its pure strain exposure. Secondly, based on LTMSE, the prototype of the DFTS is established. In terms of this sensor, a high sensitivity and stiffness can be achieved because fulcrums are separated from the lever-arm. Lastly, the method proposed in this paper has been validated by simulation and experimental results. Although the presented DFTS has been tested in one-axis torque measurement, it should be noted that the LTMSE underlying this sensor may also be applicable in multi-axial force measurement. Thus, in future reports, a more comprehensive performance of developed DFTS based on LTMSE within a concrete application will be tested.

## Figures and Tables

**Table 1:** Parameters of strain gauges.

Parameter	Description
Strain gauge type	BEM120-1AA-S-X30
Resistance	120 ± 1 Ω
Gauge factor	2.0 ± 1%
Carrier size	3.0 × 2.0 mm^2^

**Table 2:** The performance of DFTS.

Parameter	Linearity (%FS)	Hysteresis (%FS)	Repeatability (%FS)	Resolution (Nm)
DFTS	0.28	0.9	0.4	0.002
